# Supplementary material for: Adjuvant oncolytic virotherapy for personalized anti-cancer vaccination
Source: Nat Commun. 2021 May 11;12:2626. doi: 10.1038/s41467-021-22929-z (PMC8113265; doi:10.1038/s41467-021-22929-z)
Supplement: Supplementary file 1 — Supplementary Information [file 41467_2021_22929_MOESM1_ESM.pdf]

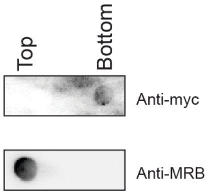

Supplementary Figure 1 Peptides do not adhere to MRB.

Dot blot analysis of retentate and filtrate fractions of MRB mixed with myc peptides and filtered using 50kDa cutoff filters. The top fraction was retained in the filter and the bottom fraction is the filtrate. Membranes were probed with MRB and myc-specific antibodies. This experiment was repeated twice. Source data are provided as a Source Data file.

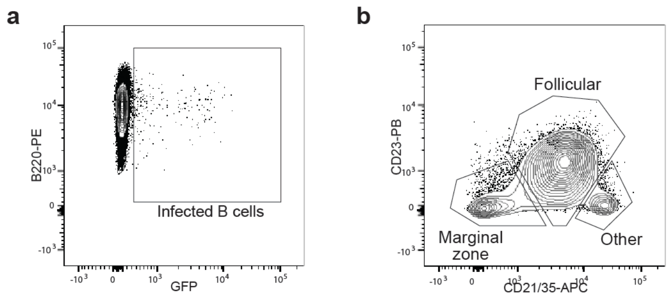

Supplementary Figure 2. Gating strategy used in Fig.1 F-G.

(**A**) Dot plot showing the GFP+ signal detected from B220$^+$ cells (pre-gated on CD19$^+$ splenocytes) from mice injected IV with MRB-GFP for 1.5 and cultured for 4.5h. (**B**) Gating strategy used to identify marginal zone, follicular and other B cell subsets based on CD21/35 and CD23 expression of CD19$^+$, B220$^+$ splenocytes from a control mouse.

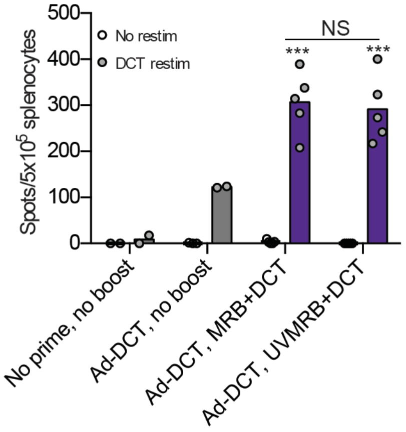

Supplementary Figure 3. Non-replicating MRB is effective when used as a vaccination adjuvant.

IFNγ ELISPOT analysis of splenocytes from mice primed with Ad-DCT and boosted with MRB+DCT or irradiated MRB+DCT (from left to right; n=2, 3, 5 and 5). The statistical analyses refer to the comparison between the *ex-vivo* “No restim” and “DCT restim” conditions. NS: $p>0.05$, *: $p<0.05$, ***: $p<0.001$ (unpaired two-tailed t-test). Source data are provided as a Source Data file. Exact p values can be found in the Source Data.

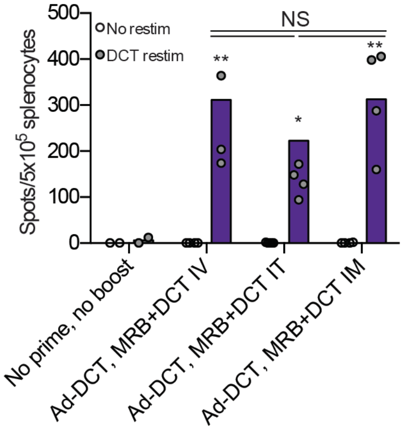

Supplementary Figure 4. MRB can be administered as a vaccination adjuvant IV, IT and IM.

IFNγ ELISPOT of splenocytes from mice primed with Ad-DCT and boosted with MRB+DCT peptide using different routes of administration (IV, IT or IM) (from left to right; n=2, 4, 5 and 4). Unless indicated otherwise, the statistical analyses refer to the comparison between the corresponding *ex-vivo* “No restim” and “DCT restim” conditions. NS: $p>0.05$, *: $p<0.05$, **: $p<0.01$ (unpaired two-tailed t-test). Source data are provided as a Source Data file. Exact p values can be found in the Source Data.

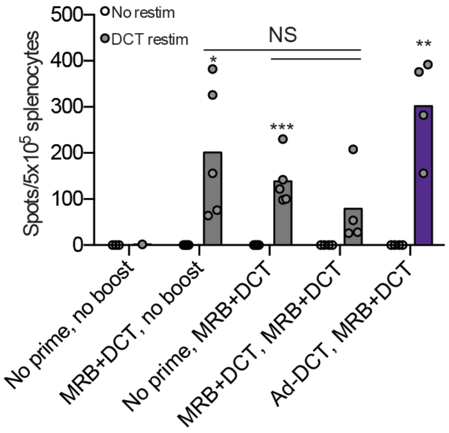

Supplementary Figure 5. MRB is less effective when used as a vaccine adjuvant for both priming and boosting.

IFNγ ELISPOT analysis of splenocytes from B16F10 SC tumor-bearing mice primed and/or boosted with MRB co-administered with DCT peptide (from left to right; n=3, 5, 5, 4 and 4). Unless indicated otherwise, the statistical analyses refer to the comparison between the corresponding *ex-vivo* “No restim” and “DCT restim” conditions. NS: $p>0.05$, *: $p<0.05$, **: $p<0.01$, ***: $p<0.001$ (unpaired two-tailed t-test). Source data are provided as a Source Data file. Exact p values can be found in the Source Data.

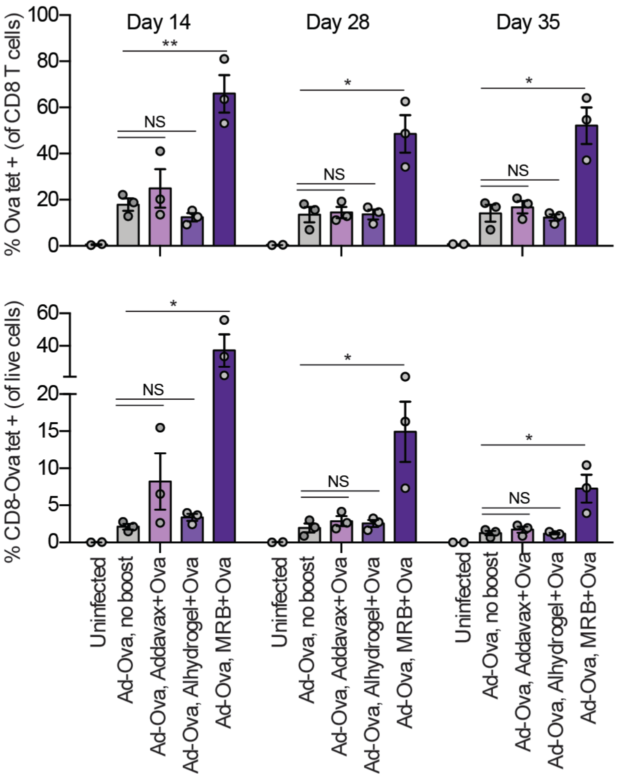

Supplementary Figure 6. MRB is better at boosting CD8-mediated T cell immune responses compared to other vaccine adjuvants used in patients.

Flow cytometry analysis of blood cells from tumor-free C57BL/6 mice primed with Ad-Ova and boosted with Addavax, Alhydrogel or MRB co-administered with Ova 7 days later (from left to right; n=2, 3, 3 and 3 (same for all time points)). Samples were collected on days 14, 28 and 35. Bar chart showing the percentage of Ova-tetramer^{+} cells (within the CD8^{+} live cell population) (top panel) or of live cells that are CD8^{+}, Ova-tetramer^{+}. NS: $p>0.05$, *: $p<0.05$ (unpaired two-tailed t-test). Source data are provided as a Source Data file. Exact p values can be found in the Source Data.

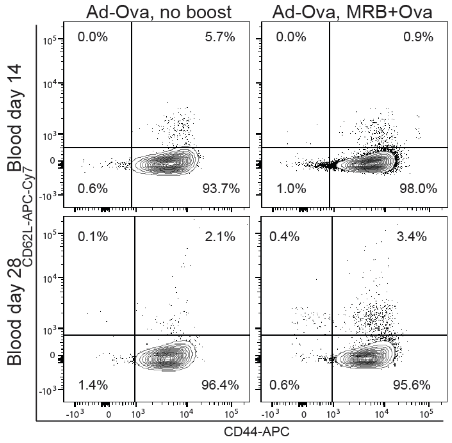

Supplementary Figure 7. Memory cell populations generated by MRB+peptide boost.

Flow cytometry analysis of CD44 and CD62L expression of $CD8^+$, Ova-$tetramer^+$ blood cells collected from tumor-free mice primed with Ad-Ova (IM) and boosted with MRB co-administered with Ova peptide on day 7. Complementary data to Fig. 1G.

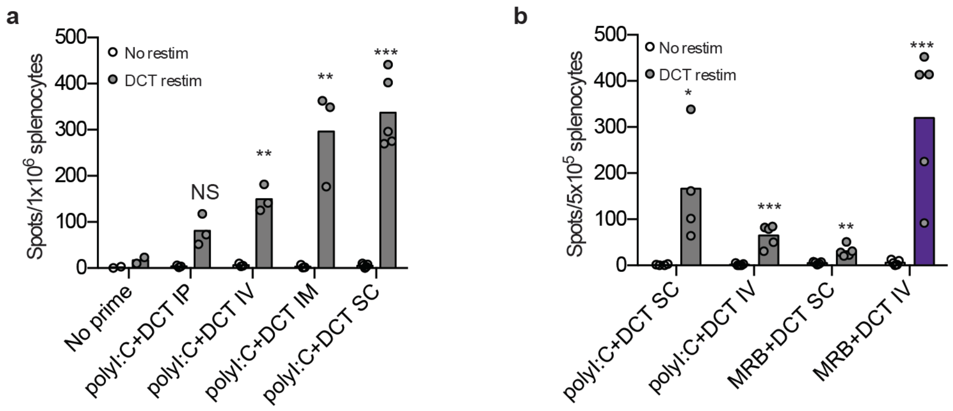

Supplementary Figure 8. PolyI:C is most effective when administered IM or SC and MRB is most effective when administered IV.

IFNγ ELISPOT analyses of splenocytes from mice immunized with (**A**) polyI:C co-administered with DCT peptide following different routes of administration (IP, IV, IM or SC) (from left to right; n=2, 3, 3, 3 and 5)or (**B**) polyI:C or MRB co-administered with DCT SC vs IV (from left to right; n=4, 5, 5 and 5). The statistical analyses refer to the comparison between the corresponding *ex-vivo* “No restim” and “DCT restim” conditions. NS: $p>0.05$, *: $p<0.05$, **: $p<0.01$, ***: $p<0.001$ (unpaired two-tailed t-test). Source data are provided as a Source Data file. Exact p values can be found in the Source Data.

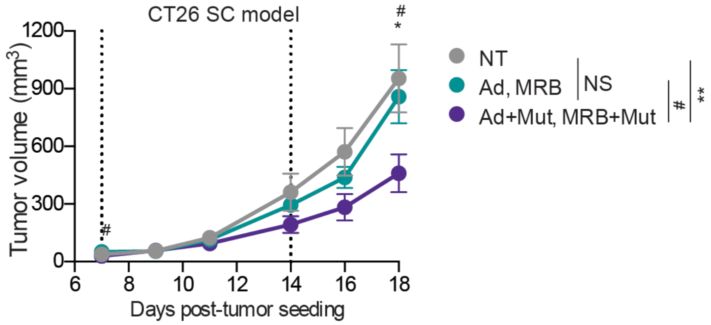

Supplementary Figure 9. The adjuvant OV prime-boost vaccine controls tumor growth.

Tumor growth curves of CT26 SC tumors from Figs. 5 E) and F). Mice were treated on days 7 and 14 post-tumor seeding with Ad and MRB or Ad and MRB co-administered with CT26Mut20, CT26Mut27 and CT26Mut37 (n=10). Data are presented as mean values +/- SEM. NS: p>0.05, ***: p<0.001 (unpaired multiple two-tailed t-test). Source data are provided as a Source Data file. Exact p values can be found in the Source Data.

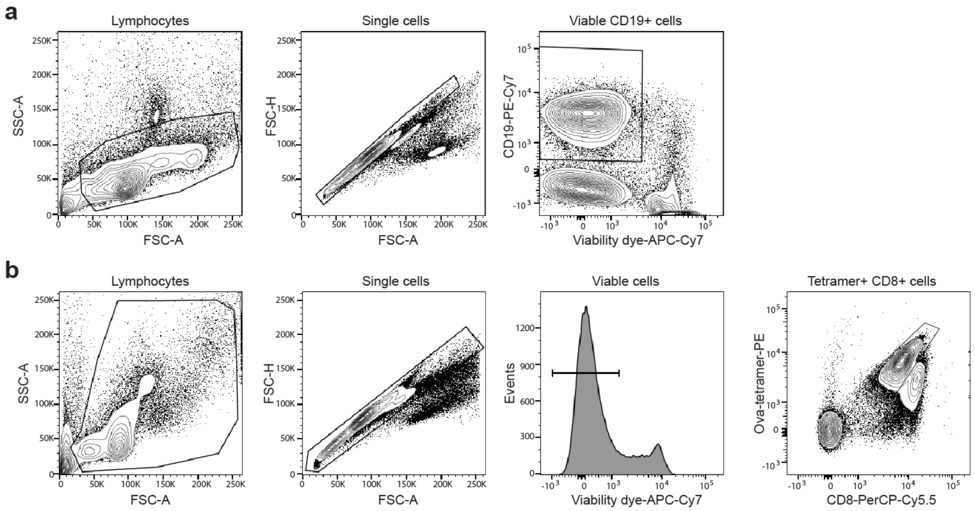

Supplementary Figure 10. Gating strategies used in this study.

Gating strategies used for (**A**) splenic B cells and (**B**) blood CD8 T cells. Gates are shown in order from left to right. The final gates and populations are shown in the main figures.
