# Supplementary material for: Adjuvant oncolytic virotherapy for personalized anti-cancer vaccination
Source: Nat Commun. 2021 May 11;12:2626. doi: 10.1038/s41467-021-22929-z (PMC8113265; doi:10.1038/s41467-021-22929-z)
Supplement: Supplementary file 3 — Reporting Summary [file 41467_2021_22929_MOESM3_ESM.pdf]

nature research

Corresponding author(s): Marie-Claude Bourgeois-Daigneault

Last updated by author(s): Mar 15, 2021

Reporting Summary

Nature Research wishes to improve the reproducibility of the work that we publish. This form provides structure for consistency and transparency in reporting. For further information on Nature Research policies, see our Editorial Policies and the Editorial Policy Checklist.

Statistics

For all statistical analyses, confirm that the following items are present in the figure legend, table legend, main text, or Methods section.

n/a	Confirmed	
☐	☒	The exact sample size (*n*) for each experimental group/condition, given as a discrete number and unit of measurement
☐	☒	A statement on whether measurements were taken from distinct samples or whether the same sample was measured repeatedly
☐	☒	The statistical test(s) used AND whether they are one- or two-sided<br>*Only common tests should be described solely by name; describe more complex techniques in the Methods section.*
☒	☐	A description of all covariates tested
☒	☐	A description of any assumptions or corrections, such as tests of normality and adjustment for multiple comparisons
☐	☒	A full description of the statistical parameters including central tendency (e.g. means) or other basic estimates (e.g. regression coefficient) AND variation (e.g. standard deviation) or associated estimates of uncertainty (e.g. confidence intervals)
☐	☒	For null hypothesis testing, the test statistic (e.g. *F*, *t*, *r*) with confidence intervals, effect sizes, degrees of freedom and *P* value noted<br>*Give P values as exact values whenever suitable.*
☒	☐	For Bayesian analysis, information on the choice of priors and Markov chain Monte Carlo settings
☒	☐	For hierarchical and complex designs, identification of the appropriate level for tests and full reporting of outcomes
☒	☐	Estimates of effect sizes (e.g. Cohen's *d*, Pearson's *r*), indicating how they were calculated

Our web collection on statistics for biologists contains articles on many of the points above.

Software and code

Policy information about availability of computer code

Data collection	No software was used
Data analysis	Flow cytometry data: FlowJo version 10.5.3. All graphs: Prism version 6.0e

For manuscripts utilizing custom algorithms or software that are central to the research but not yet described in published literature, software must be made available to editors and reviewers. We strongly encourage code deposition in a community repository (e.g. GitHub). See the Nature Research guidelines for submitting code & software for further information.

Data

Policy information about availability of data

All manuscripts must include a data availability statement. This statement should provide the following information, where applicable:

- Accession codes, unique identifiers, or web links for publicly available datasets
- A list of figures that have associated raw data
- A description of any restrictions on data availability

All data generated during and/or analysed during this study are available from the corresponding author on reasonable request.

Field-specific reporting

Life sciences study design

All studies must disclose on these points even when the disclosure is negative.

Sample size	The sample size was chosen based on previous experiment we performed in the lab. By experience, we obtained reproducible data using 3-5 mice per group for the animal studies and only 2 for controls as the background is very low. We have previously published similar analyses using these sample sizes (Aitken et. al, J. Immunother 2018).
Data exclusions	No data was excluded.
Replication	All findings were reproducible. Experiments were independently performed the following number of times: Fig. 1b (2), 1c (2), 1d (2), 1e (2), 1f (2), 1g (2), 2b (2), 2c (4), 2d (3), 2e (1), 2f (1, 2 experiments combined), 3b (2), 3c (2), 3d (2), 3e (2), 3f (2), 3g (2), 3h (2), 3i (2), 4b (4), 4c (2), 3d (1), 4e (1, 2 experiments combined), 5b (1), 5c (1), 5d (1), 5e (2), 5f (2), 6b (1, 2 experiments combined), 6c (1), 6d (2), 6e (1, 2 experiments combined), 6f (1), 6g (1), 7b (2), 7c (2), S1 (2), S2a (2), S2b (2), S3 (1), S4 (1), S5 (1), S6 (1), S7 (2), S8a (1), S8b (1), S9 (2).
Randomization	Given that we inject virus to our animals and to avoid contamination, we keep the different animal groups in separate cages. The cages were allocated randomly.
Blinding	The investigators were not blinded because the animals were injected with viruses and not knowing what the user is exposed to is not recommended.

Reporting for specific materials, systems and methods

We require information from authors about some types of materials, experimental systems and methods used in many studies. Here, indicate whether each material, system or method listed is relevant to your study. If you are not sure if a list item applies to your research, read the appropriate section before selecting a response.

Materials & experimental systems

n/a	Involved in the study	
☐	☒	Antibodies
☐	☒	Eukaryotic cell lines
☒	☐	Palaeontology and archaeology
☐	☒	Animals and other organisms
☒	☐	Human research participants
☒	☐	Clinical data
☒	☐	Dual use research of concern

Methods

n/a	Involved in the study	
☒	☐	ChIP-seq
☐	☒	Flow cytometry
☒	☐	MRI-based neuroimaging

Antibodies

Antibodies used	Rat monoclonal anti-mouse CD62L (Clone MEL-14, APC-Cy7, eBioscience #47-0621-82, 1:300); Rat monoclonal anti-mouse TNF-alpha (Clone MP6-XT22, APC, eBioscience #17-7321-82, 1:250); Rat monoclonal anti-mouse IFN-gamma (Clone XMG1.2, FITC, eBioscience #11-7311-41, 1:250); Rat monoclonal anti-mouse CD8 (Clone 53-6.7, PerCPCy5.5, eBioscience #45-0081-82, 1:300); Rat monoclonal anti-mouse CD127 (Clone A7R34, BV421, BioLegend #135024, 1:100), Hamster monoclonal anti-mouse KLRG1 (Clone 2F1, FITC, eBioscience #11-5893-82, 1:300); Rat monoclonal anti-mouse CD44 (Clone IM, APC, eBioscience #17-0441-83, 1:300); tetramer H-2Kb/OVA257 (Tetramer Baylor College of Medicine, PE, 1:100); Fixable Viability Dye eFluor 506 (#65-0866-14, eBioscience, 1:750); Fixable Viability Dye eFluor 780 (#65-0865-14, eBioscience, 1:1000); Rat anti-mouse CD21/CD35 (clone 7E9, APC, BioLegend #123411, 1:100); anti-mouse CD23 (clone B3B4, eFluor450, BioLegend #48-0232-82, 1:300); Rat anti-mouse B220 (Clone RA3-6B2, PE, eBioscience, 1:300); Rat anti-mouse CD19 (Clone ID3, PE-Cy7, eBioscience #25-0193-82, 1:300); Mouse anti myc (Clone 9B11, cat#2276, Cell signaling technology, 1:1000); Rabbit anti-MRB (Homemade, 1:3000); Rat IgG2ak anti-mouse CD8a (clone 53.67, Leinco cat# C2848, 100µg/mouse); Peroxydase-coupled goat anti-mouse (Invitrogen, #32230, 1:1000); Peroxydase-coupled goat anti-rabbit (Invitrogen, #65-6120, 1:1000).
Validation	CD8: Staining of C57BL/6 splenocytes with 0.125 µg of Rat IgG2a K Isotype Control PerCP-Cyanine5-5 or 0.125 µg of Anti-Mouse CD8a PerCP-Cyanine5-5. Cells in the lymphocyte gate were used for analysis.<br><br>CD44: Staining of C57BL/6 splenocytes with staining buffer (autofluorescence) or 0.03 µg of Anti- Mouse CD44 APC. Total viable cells were used for analysis.<br><br>KLRG1: Staining of C57Bl/6 splenocytes with Anti-Mouse NK1-1 APC and 0.125 µg of Golden Syrian Hamster IgG Isotype Control FITC or 0.125 µg of Anti-Mouse KLRG1 FITC. Total viable cells were used for analysis.<br><br>CD127: C57BL/6 mouse splenocytes were stained with CD3ε FITC and CD127 (clone A7R34) Brilliant Violet 421 or rat IgG2a, κ Brilliant Violet 421™ isotype control.<br><br>CD62L: Staining of BALB/c splenocytes with Anti-Mouse CD3e FITC (and 0.06 µg of Rat IgG2a K Isotype Control APC-eFluor 780 or 0.06

µg of Anti-Mouse CD62L (L-Selectin) APC-eFluor 780. Total viable cells were used for analysis.

IFNg: Mouse splenocytes were cultured in the presence of Protein Transport Inhibitors (500X) or Cell Stimulation Cocktail (plus protein transport inhibitors, 500X) for 5 hours. Cells were fixed and permeabilized with the IC Fixation and Permeabilization Buffer Set and protocol followed by intracellular staining with CD4 (clone RM4-5), NK1.1 (clone PK136) and IFN gamma (clone XMG1.2). Cells in the CD4+ or NK1.1+ gates were used for analysis.

TNFa: Intracellular staining of Mouse Cytokine Positive Control Cells with Anti-Mouse CD4 PE and 0.125 µg of Rat IgG1 K Isotype Control APC or 0.125 µg of Anti-Mouse TNF alpha APC.

CD19: Staining of BALB/c splenocytes with Anti-Human/Mouse CD45R (B220) FITC and 0.125 µg of Rat IgG2a K Isotype Control PE-Cyanine7 or 0.125 µg of Anti-Mouse CD19 PE-Cyanine7. Cells in the lymphocyte gate were used for analysis.

B220: Staining of mouse splenocytes and bone marrow cells. As expected based on known relative expression patterns, CD45R (B220) clone RA3-6B2 stains subsets in both the splenocytes gate and the bone marrow lymphoid gate but not the bone marrow myeloid gate. Details: Balb/c bone marrow cells (left) and splenocytes (middle) were surface stained with CD45R (B220, clone RA3-6B2) followed by staining with 7-AAD. Viable bone marrow cells in the lymphoid and myeloid gates and viable splenocytes were used for analysis.

CD21/35: C57BL/6 mouse splenocytes stained with 7E9 APC

CD23: Staining of BALB/c splenocytes with Anti-Human/Mouse CD45R (B220) APC and 0.25 µg of Rat IgG2a K Isotype Control eFluor 450 or 0.25 µg of Anti-Mouse CD23 eFluor 450. Total viable cells were used for analysis.

Viability eFluor780: BALB/c thymocytes were uncultured or cultured overnight at 37°C and then stained with Fixable Viability Dye eFluor 780. Total cells were used for analysis.

Viability eFluor 506: Staining of C57Bl/6 thymocytes cultured overnight with staining buffer (autofluorescence) or Fixable Viability Dye eFluor506. Total cells were used for analysis.

Ova-tetramer: Staining blood cells from C57Bl/6 vs OT-I (Ova-specific CD8 T cells transgenic) mice.

Mouse anti myc (Clone 9B11, cat#2276, Cell signaling technology): Western blot analysis of cell extracts expressing Myc-tagged protein or control cell extracts.

Rabbit anti-MRB (Homemade); Western blot analysis of cells infected or not with MRB virus (Bourgeois-Daigneault et. al., Science Translational Medicine, 2018).

Rat IgG2ak anti-mouse CD8a (clone 53.67, Leinco); Competes with anti-CD8 antibody clone 5H10-1 for binding to thymocytes.

Peroxydase-coupled goat anti-mouse (Invitrogen, #32230, 1:1000); Western blot analysis performed on whole cell extracts (30 µg lysate) of HeLa and Jurkat probed with Anti-SOD1 Mouse Monoclonal Antibody.

Peroxydase-coupled goat anti-rabbit (Invitrogen, #65-6120, 1:1000); Western blot analysis performed on whole cell extracts (30 µg lysate) of HeLa and PC-3 probed with Anti-PRDX6 recombinant Rabbit Monoclonal Antibody.

Eukaryotic cell lines

Policy information about cell lines

Cell line source(s)	CT26, B16F10, Vero, 293X, U2OS and HeLa cells were purchased from ATCC.
Authentication	None of the cell lines were authenticated
Mycoplasma contamination	All cell lines were negative for mycoplasma.
Commonly misidentified lines (See ICLAC register)	None

Animals and other organisms

Policy information about studies involving animals; ARRIVE guidelines recommended for reporting animal research

Laboratory animals	6-8 week old female C57BL/6 and BALB/c mice were purchased from Charles River. Mice were housed in a room with controlled temperature of 22°C and humidity of 45–65% under a 12-h light and 12-h dark cycle.
Wild animals	This study did not involve wild animals.
Field-collected samples	This study did not involve samples collected from the field.

Ethics oversight	The University of Ottawa Animal Care Committee (ACC) approved all animal work.

Note that full information on the approval of the study protocol must also be provided in the manuscript.

Flow Cytometry

Plots

Confirm that:

- [x] The axis labels state the marker and fluorochrome used (e.g. CD4-FITC).
- [x] The axis scales are clearly visible. Include numbers along axes only for bottom left plot of group (a 'group' is an analysis of identical markers).
- [x] All plots are contour plots with outliers or pseudocolor plots.
- [x] A numerical value for number of cells or percentage (with statistics) is provided.

Methodology

Sample preparation	For flow cytometry analysis of blood, blood was collected from the tail vein and added into PBS supplemented with 2% FBS, 5mM EDTA and 0.02% sodium azide. Red blood cells were lysed with ACK lysis buffer before staining.
Instrument	Fortessa LSRII
Software	FlowJo version 10
Cell population abundance	The percentage of each population is indicated in the corresponding graphs.
Gating strategy	The gating strategies are shown in supplemental figure 10. The negative populations were set based on the signal obtained for negative controls. For tetramer gating, the gates were set based on the signal optained for non vaccinated animals.

- [x] Tick this box to confirm that a figure exemplifying the gating strategy is provided in the Supplementary Information.
